# The Diagnosis and Management of Urosymphyseal Fistula with Pubic Osteomyelitis

**DOI:** 10.1007/s11934-025-01293-1

**Published:** 2025-09-30

**Authors:** Claire Haas, Amir Feinberg, George E. Koch, Hiren V. Patel

**Affiliations:** 1 Department of Urology, The Ohio State University Wexner Medical Center, 915 Olentangy River Road, Suite 3117, Columbus, OH USA; 2 Department of Urology, University of Washington, Seattle, WA USA

**Keywords:** Urosymphyseal fistula, Pubic osteomyelitis, Reconstructive urology

## Abstract

**Purpose of Review:**

Urosymphyseal Fistula (USF) is a rare, often debilitating condition in which the urinary tract fistulizes with the pubic bone, resulting in recurrent pubic osteomyelitis (POM) from urinary tract seeding. These injuries commonly arise in the setting of pelvic radiation and are generally preceded by instrumentation of the lower urinary tract for obstruction. USFs tend to be recognized only after patients fail to recover from multiple lower urinary tract infections, resulting in repeat hospital readmissions. While fistula closures with urinary tract reconstruction have been described, treatment of USF is often complex and is best treated via urinary diversion with pubic bone debridement in the setting of preoperative optimization and long-term postoperative antibiotic regimens. The general understanding of USF presentation, work-up, and management is largely based on retrospective reviews with small sample sizes and case reports. Here we review the etiology, diagnosis, and considerations when providing care for this complex condition and propose a management algorithm to guide care.

**Recent Findings:**

Recent data on USF remain limited, with the literature dominated by small, retrospective case series and single-institution reviews. Most published studies report on cohorts of fewer than 30 patients, often focusing on men with a history of pelvic radiotherapy and subsequent urethral manipulation for prostate cancer. There is a notable absence of formal guidance from professional societies regarding standardized preoperative evaluation, diagnostic criteria, antimicrobial regimens, surgical techniques, or follow-up protocols for this condition. The literature published in the past 18 months continues to be sparse, with fewer than 10 new publications identified, underscoring the rarity of the condition and the ongoing need for multicenter studies and consensus guidelines.

**Summary:**

The presentation of USF can be quite subtle initially, and thus its diagnosis is nuanced and requires providers maintain a low threshold for further evaluation in high-risk patients with pelvic pain and refractory urinary tract infections. Here, we propose a comprehensive management pathway, informed by the current data, data from relevant related disease processes and institutional experience, that begins with contrast-enhanced imaging, preoperative medical optimization, and early involvement of infectious disease and surgical subspecialists to support perioperative and intraoperative planning and management. Ultimately, long durations of culture-directed antibiotics as well as surgical debridement with cystectomy, pubectomy, and urinary diversion is the most definitive method for cure of this condition. Long-term, high-volume studies on management and outcomes in these patients have yet to be performed.

**Supplementary Information:**

The online version contains supplementary material available at 10.1007/s11934-025-01293-1.

## Introduction

Urosymphyseal fistula (USF) with pubic osteomyelitis (POM) is a rare but devastating complication of lower urinary tract radiation and instrumentation, often in the setting of prostate cancer therapy. This condition most often arises following radiotherapy and urologic endoscopic interventions for lower urinary tract obstruction, presenting with debilitating pelvic pain, difficulty with ambulation, and recalcitrant urinary tract infections [1, 2]. The diagnosis and management of USF with subsequent POM remains complex and institution-dependent with optimal treatment typically requiring a multidisciplinary approach involving collaboration between urology, infectious disease, plastic surgery, and orthopedic surgery. Definitive treatment generally necessitates both prolonged medical therapy and surgical intervention, with surgical strategy tailored to the extent of infection, underlying etiology, and patient comorbidities.

## Etiology

USF is a rare but serious complication more often occurring in patients treated for prostate cancer with radiation therapy in combination with surgical interventions such as radical prostatectomy or endoscopic procedures [3]. Given this etiology, the condition is generally seen in men with an average age of 67–69 [1, 4]. Radiotherapy is a significant predisposing factor, with studies indicating that up to 93% of USF cases occur in patients who have received pelvic radiation for prostate cancer [4–6]. In patients who undergo a radical prostatectomy in the context of prior radiotherapy, this surgical intervention can act as a significant insult, leading to the formation of a fistula in the previously radiated tissue [1]. However, the more common scenario involves endoscopic interventions, such as serial dilation, balloon dilation, and urethrotomy with cold knife or laser, to treat radiation-induced posterior urethral stenosis or radiation associated vesicourethral anastomotic stenoses [4]. This iatrogenic trauma of irradiated tissue can lead to poorly healing urethral injuries which result in in the formation of a fistulous tract between urethra and pubic symphysis [1]. These minimally traumatic procedures, even when performed in the setting of uninfected urine, can precipitate the development of USF and subsequent POM [5].

While USF is most often described in men with a history of prostate radiotherapy and urethral manipulation, it has been rarely described in women as well, with one case series describing four women with USF, only two of which had a history of any pelvic radiotherapy. Those who did not have prior radiation did have a history of neurogenic bladder managed with chronic indwelling foley [7].

The infectious etiology of POM associated with USF is well-documented, however reported pathogens vary. A single, isolated organism may be the causative pathogen in POM; however, polymicrobial infections are also commonly seen [6]. One review of 33 patients with POM in the setting of USF found Escherichia coli present in 25.8% of urine cultures with Pseudomonas the second most common in 12.9% of urine cultures [4]. In patients with polymicrobial infections, positive bone cultures are found in most patients undergoing extirpative surgery for USF, with common organisms including Candida (22%), Enterococcus (18%), and Pseudomonas (10%) [8]. Important to note is the lack of clear concordance between preoperative urine cultures and intraoperative bone cultures. In one retrospective analysis investigating the management of USF and associated POM, the concordance rate between organisms identified in pre-operative urine cultures and surgically collected bone cultures was approximately 60% however another retrospective analysis notes a 95.5.% discordance rate between the two cultures [4, 9].

## Diagnosis

### Clinical Presentation

**Fig. 1:**
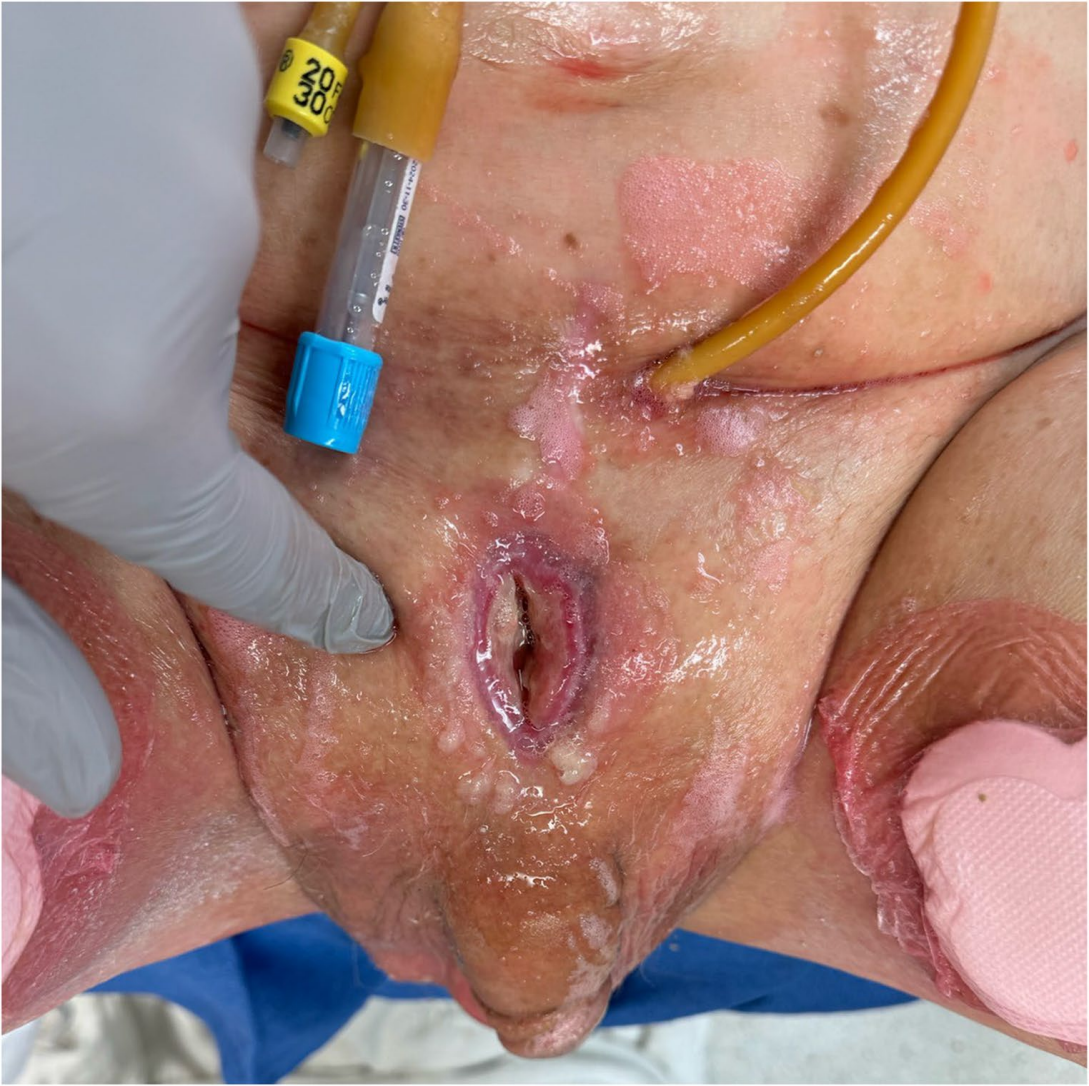
Urethrocutaneous fistula in patient undergoing surgical intervention for pubic osteomyelitis and urosymphyseal fistula at our institution

Patients typically present with a constellation of symptoms that significantly impact their quality of life. The most common presenting symptoms include debilitating pelvic pain, which is often exacerbated by ambulation, and difficulty walking. This pain is frequently described as severe and persistent, leading to a waddling gait or immobility [3, 10]. Additionally, patients often report recurrent urinary tract infections (UTIs), skin infections, or cutaneous drainage which are indicative of the underlying fistulous connection between the urinary tract and the pubic symphysis (Fig. 1) [2]. These symptoms are often non-specific and can be mistaken for other conditions like osteitis pubis or chronic prostatitis [11]. Coupled with the chronic, progressive nature of radiation-induced injury, symptoms often do not become apparent until years after the initial treatment leading to significant diagnostic challenges [2, 5]. Inguinal lymphadenopathy is an uncommon finding, present in just 4% of patients in one study [12]. In a retrospective study of 33 patients treated for USF who underwent simple cystectomy, approximately 94% of patients had a history of prior pelvic external beam radiotherapy or brachytherapy, however median time of diagnosis was 11 years after cancer treatment. About 90% of those patients had documented intermittent catheterization or endoscopic bladder outlet procedure, with median diagnosis of USF about 10 months after urethral manipulation [4]. One retrospective study notes rates of prior endoscopic procedure to be as high as 100% in USF patients, while another reports 60% of these patients having a history of multiple endoscopic urethral procedures prior to USF diagnosis [1, 9].

### Diagnostic Workup

As most patients will present acutely due to pain or infectious symptoms to an ED or urgent care, they typically will have up-to-date labs when the urologist is engaged. Given the infectious nature of the condition, leukocytosis is common, but not universal. Inflammatory markers, including C-reactive protein (CRP) and erythrocyte sedimentation rate (ESR), are often elevated in patients with USF and POM, but these laboratory values are inconsistent and nonspecific in this setting [13]. A urinalysis will show hematuria and pyuria and be consistent with a urinary tract infection, leading to collection of a urine culture, which aids in pathogen identification. Blood cultures can also be useful for identifying the pathogens involved in the setting of sepsis. Cultures from associated abscesses may also help identify causative organisms but are often incongruent with pathogens growing in the deeper pelvic structures [3, 8]. As mentioned previously, data suggests a mediocre congruence rate between organisms identified in pre-operative urine cultures and surgically collected bone cultures. While urine cultures are not irrelevant and should be covered for, this underscores the importance of broad antimicrobial coverage initially while all cultures, including bone, are speciated [8, 10].

Cross-sectional imaging is essential in accurately diagnosing USF. Plain radiographs are generally insufficiently sensitive to detect osteomyelitis; however, they may be useful for excluding alternative diagnoses such as fracture, dislocation, or malignancy [14, 15]. Computed tomography (CT) with intravenous contrast is more sensitive than X-ray and may detect synovitis, inflammation, abscesses, gas, and vascular complications [15, 16]. The use of IV contrast, nonetheless, does not significantly improve the diagnostic sensitivity for acute osteomyelitis itself [15]. There is evidence that voiding cystourethrography and urethrography can be helpful in identifying USF, particularly when distal lower tract is involved [17].

**Fig. 2:**
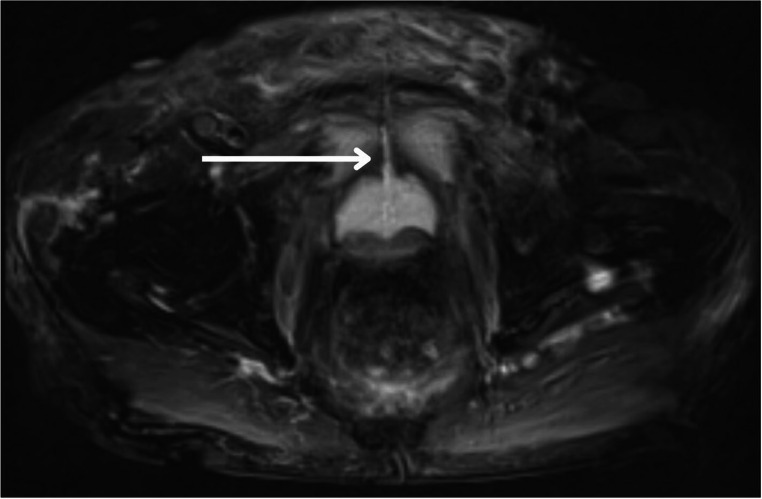
Pelvic MRI demonstrating fistulous tract between urethra and pubic symphysis

**Fig. 3:**
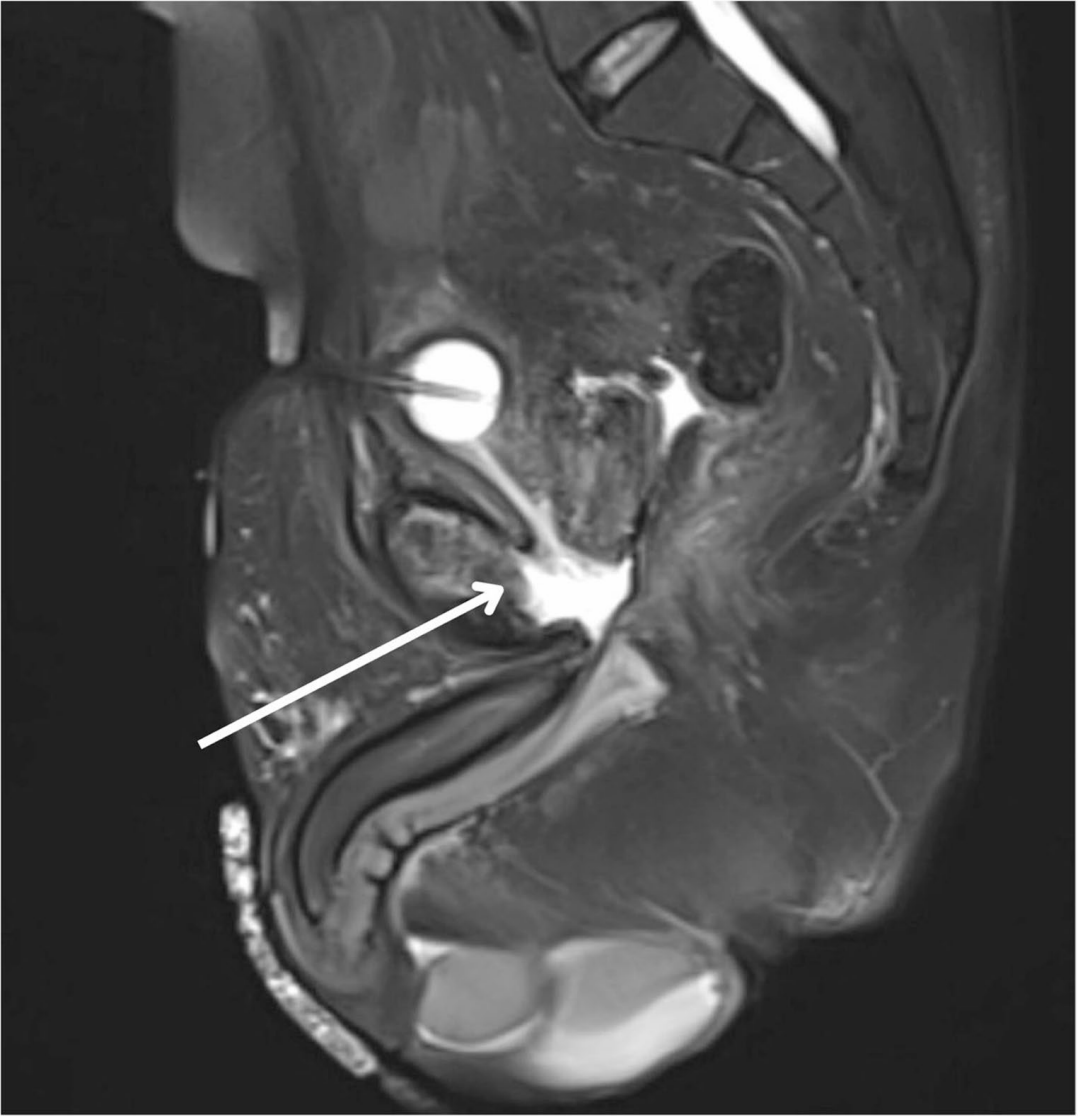
Sagittal view of pelvic MRI demonstrating fistulous tract between urethra and pubic symphysis

**Fig. 4:**
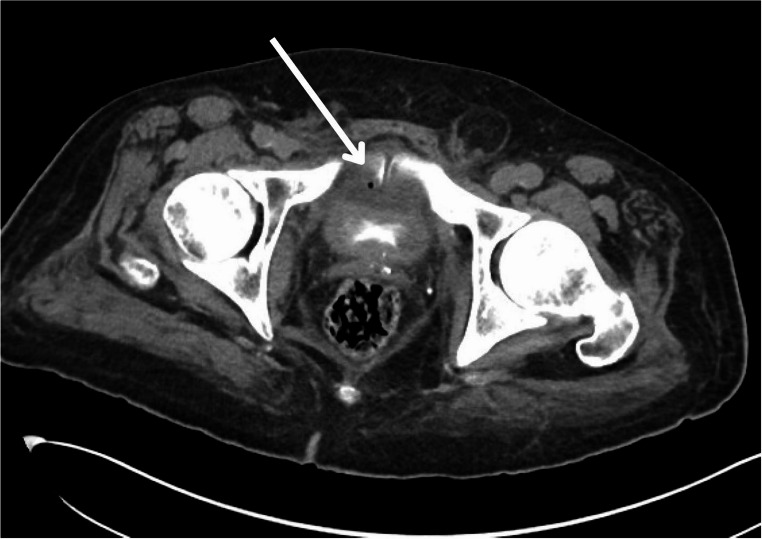
CT Urogram with air adjacent to bladder at the level of the pubic symphysis- an indicator of potential infection and fistula

Ultimately, magnetic resonance imaging (MRI) with contrast remains the most sensitive modality for identifying early stages of osteomyelitis and is the gold standard imaging modality for evaluating osteomyelitis of any location [18, 19]. The use of contrasted MRI or CT with nephrogenic and excretory phases for the diagnosis of USF is described in literature and used at our institution (Figs. 2, 3 and 4). Use of MRI is favored due to its optimal imaging of soft tissue structures and is characterized by increased signal in T2-weighted images and decreased signal in T1-weighted images of the pubic rami [19, 20]. Extent of soft tissue involvement of POM is best evaluated with this imaging modality and may help guide surgical planning in the case of USF [4].

While imaging alone can diagnosing USF, a presumptive diagnosis of POM can often be made based on clinical presentation, including symptoms, risk factors, and supportive imaging findings, particularly when accompanied by positive urine or wound cultures. Medical and surgical management may be initiated based on high enough suspicion from these findings, despite, definitive diagnosis of POM requiring surgically obtained tissue culture from the pubic symphysis. The highest yield for accurate specimen collection typically occurs at the time of surgical debridement. Given the source of POM in USF fistula is the connection between the genitourinary tract and the bone, bone biopsy and culture are often collected at the time of fistula takedown. Nevertheless, the role of biopsy remains controversial, with some series reporting culture positivity rates as low as 35% in confirmed cases of POM [21].

## Management

### Medical Management

Medical management for USF starts at the time of presentation and includes patient optimization prior to surgery, during hospitalization, and postoperatively with antimicrobials. Given the need for long-term intravenous antibiotics in most cases, consultation with infectious disease specialists at the time of initial diagnosis of POM in the setting of USF is crucial for optimal management of long-term antibiotic therapy. These patients will often require multiple admissions for infectious sequelae prior to definitive surgery, and thus the complexity and severity of these infections almost always require the expert guidance of an infectious disease physician regarding the selection and duration of antimicrobial therapy, ensuring that treatment is both effective and tailored to the specific pathogens involved [22].

While targeted antibiotic therapy is the ideal of medical management in POM secondary to USF, bacterial and fungal growth from superficial cultures or cultures from the urinary tract do not correlate strongly enough with the final pathologic specimens to drive antimicrobial selection without an expert [23]. Further, persistent contamination of the bone via its connection with the urinary tract will not allow for complete microorganism clearance without debridement. Therefore, initial antibiotic choices are made empirically. Finally, bone cultures and biopsies can be sent during pubic bone debridement for more definitive antibiotic plans.

Without a culture from the site of osteomyelitis, antibiotic guidance is limited. Retrospective studies recommend broad-spectrum coverage targeting staphylococcal species, gram-negative bacilli, as well as fungal and anaerobic organisms [8, 24, 25]. Reviews of antimicrobial selection in USF and POM report a large variety of antibiotics, often agents targeting multidrug resistant species, specifically carbapenems [25]. Moreover, final bone cultures commonly grow yeast, so antifungal coverage with fluconazole can be additionally employed [3, 8]. At our institution, preoperative antibiotics may be prescribed in setting of recurrent symptomatic urinary tract infections and are directed by urine culture sensitivities. We generally stop these antibiotics 5 days before definitive extirpative surgery in order to optimize the yield of bone cultures and biopsies taken during pubic bone debridement.

Following definitive bone debridement for POM and urinary diversion for USF, the timing of antibiotic therapy is controversial. The Infectious Diseases Society of America (IDSA) recommends a minimum of 6 weeks of intravenous, broad-spectrum antibiotics for chronic osteomyelitis, with duration extended up to one year based on clinical response, imaging, and culture results [25]. However, data on osteomyelitis at all locations suggest that oral administration upon discharge following surgical debridement may be as effective in treating the infection [26, 27]. Notably, much of this data is based on the management of diabetic foot infections rather than USF and OM [27–29]. Data regarding antifungal coverage in USF is limited. When antifungal coverage is indicated our practice pattern includes continuing oral antifungals for 6 months to 12 months which is consistent with recommendations for osteomyelitis coverage at any locations [30]. Preoperative urinary diversion is essential. While there are no guidelines with regards to the preferred method of diversion, ensuring as little urine as possible is allowed to bathe the pubic bone follows the clinical principle of source control in urologic infections. Urinary diversion can thus be achieved with nephrostomy tubes, a suprapubic tube, or a urethral catheter, depending on each patient’s specific case.

Aside from disease treatment, pain management should be an additional focus. Guidance is minimal in this regard, however multiple publications detail the risk for the development of opioid-dependence in those suffering from USF long-term, suggesting the need for alternate and multimodal pain management strategies and potential for involvement of palliative care or chronic pain teams [4, 31].

### Pre-Surgical Management

In patients with a history of radiotherapy for prostate cancer, non-surgical treatments for USF are rarely successful, necessitating more definitive surgical interventions. In a retrospective review of patients with pelvic pain and associated USF following prostate radiation, 15 of 16 patients required eventual surgical intervention to manage symptoms [2].

Preoperative counseling is critical in optimizing patients with USF with POM and includes discussion of the risks of dehydration, digestive changes, wound healing complications- including wound dehiscence and infection- significant bleeding, and rarely more devastating cardiac and neurologic complications or mortality. Data from a cohort of 389 patients who underwent simple cystectomy for benign disease demonstrated a 30-day postoperative complication rate of 60.4%. The most common complications included bleeding requiring transfusion within 72 h (38.6%), wound infection (16.2%), respiratory complications (7.5%), renal complications (2.3%), wound dehiscence (2.1%), postoperative deep vein thrombosis (2.1%), and cardiovascular complications (1.5%) [32]. Literature on optimization prior to surgery is limited in the benign cystectomy population, however there have been multiple publications detailing optimization in patients undergoing radical cystectomy which we apply to the benign cystectomy population.

At our institution, patients receive nutritional counseling and are recommended to supplement their protein intake [33]. While data on preoperative nutritional optimization in USF patients undergoing simple cystectomy is limited, there is literature suggesting poor preoperative nutritional status is a predictor of postoperative surgical complications in the radical cystectomy population [34, 35]. Optimization of medical comorbidities is critical and may include management of cardiovascular disease, normalizing bowel function, and improving anemia with iron supplementation [36–38]. Smoking cessation is essential for wound healing as has previously been described in plastic and colorectal surgery patients, and patients also are encouraged to increase functional capacity preoperatively through ambulation or other exercise [33, 39–43]. Hyperbaric oxygen therapy (HBOT) can be used preoperatively to enhance tissue oxygenation, promote angiogenesis, and reduce complications—especially in patients with radiation tissue injury where it helps mitigate radiation-related damage [44]. Data supports the efficacy of HBOT in significantly alleviating symptoms of late radiation cystitis, improving patient-perceived urinary symptoms and quality of life [44, 45].

Additionally, all patients are seen by specialized wound ostomy nurses for perioperative wound care and stoma marking to help patients and caregivers become comfortable with postoperative stoma management in concordance with American Urological Association and Wound, Ostomy and Continence Nurses Society position statement which is largely based on data from colorectal surgery patients [46, 47]. In the immediate preoperative period, immunosuppressants are held to promote wound healing and minimize infection risk, and anticoagulation is held prior to surgery as able based on surgical and anesthesiology guidelines in the perioperative period to minimize perioperative bleeding respectively [48–51].

We advocate for early mobilization to avoid deconditioning and promote early recovery after surgery consistent with most enhanced recovery after surgery guidelines including those for patients undergoing radical cystectomy [52–54]. Patients are additionally counseled on appropriate postoperative expectations including the potential need for prolonged hospitalization or need for rehabilitation facility at discharge. One retrospective cohort study of 138,151 patients who underwent radical cystectomy from 2000 to 2019 reported 18% of patients ultimately were discharged to continued rehabilitation facility. Risk factors for discharge to rehabilitation facility included age and Charlson Comorbidity Index score. While data on discharge disposition in benign cystectomy patients, or even more specifically, those with USF is exceptionally limited, literature does suggest patients undergoing benign cystectomy have worse health status and American Society of Anesthesiologists scores preoperatively [55].

### Surgical Management

The surgical management of USF with POM often necessitates a multidisciplinary approach which may involve orthopedic surgeons, colorectal surgeons and plastic surgeons in addition to urologic surgeons [11]. Given the frequent history of pelvic radiation and impaired wound healing, conservative measures are typically insufficient, requiring more aggressive surgical intervention [9, 56]. In cases where the urinary bladder is extensively involved, extirpative procedures such as cystectomy or cystoprostatectomy with urinary diversion are performed. Often a supratrigonal cystectomy is preferred to avoid undue injury to the rectum, which may necessitate a permanent colostomy in the setting of prior radiation. However, while salvage vs. simple cystectomy can be debated, pubic symphyseal resection and radical debridement of necrotic tissue are essential to eradicate infection. Anterior debridement of the pubic bone is usually well tolerated and should not lead to an unstable pelvis due to the fixed ring configuration of the pelvis. However, in patients with prior pelvic fractures or posterior urethral injuries care is advised and consulting orthopedic surgery is warranted as fixation may be necessary. In addition, flap coverage of the pelvic defect is necessary and often a rectus abdominis, peritoneal or omental flap are needed to cover this area [11]. In these instances, involvement of plastic and reconstructive surgery preoperatively can assist with complex flap coverage and abdominal wall closures.

**Fig. 5:**
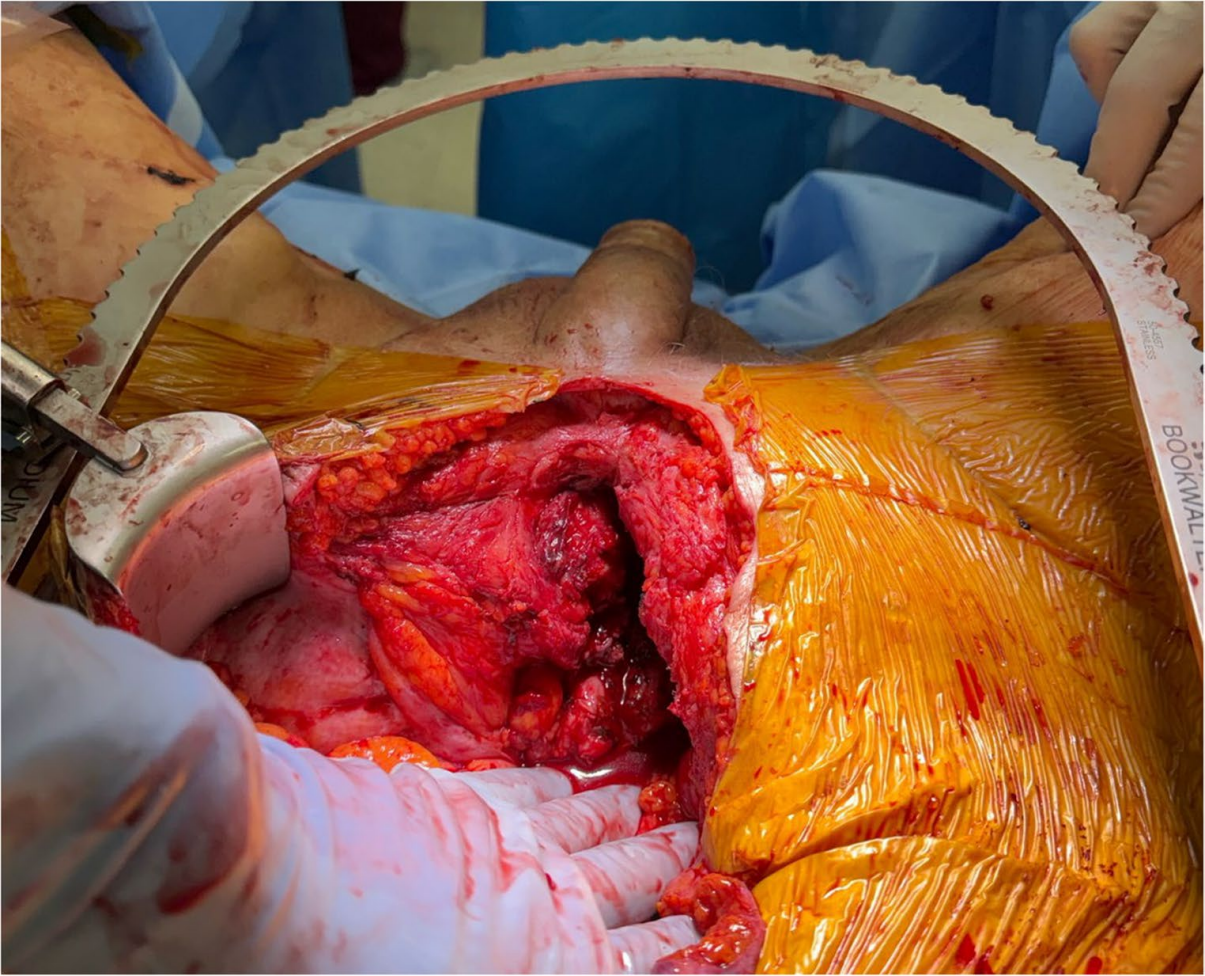
Surgical intervention of urosymphyseal fistula with pubic bone debridement

Historically, an open abdominal approach has been the standard for extensive debridement, fistula excision, and pubic symphyseal resection (Fig. 5) [5, 9]. The affected symphysis is typically excised back to healthy, bleeding tissue while the fistulous tract is completely excised with all associated cavities curetted and drained.

Orthopedic surgery may be engaged to assist with this resection. Urologic reconstructive surgeons may be comfortable performing this themselves, especially those with experience performing pubectomy during posterior urethroplasty, however it is important to consider that resection in the setting of POM may be significantly more extensive. As the pelvis is a fixed ring, resection of the pubic symphysis anteriorly should not have significant impact on stability or gait, however in patients with prior pelvic ring injuries, consideration of posterior fixation prior to anterior resection of the pubis may be considered.

Urinary diversion using either ileum or colon conduits then proceeds in the standard fashion. One study investigated the role of near-infrared fluorescence imaging (NIFI) combined with indocyanine green (ICG) in assessing ureteral tissue perfusion during open urinary diversion and showed that NIFI/ICG changed intraoperative decision-making in 63% of cases, particularly in patients with prior radiation [57]. Therefore, we have adopted the use of NIFI/ICG to help delineate healthy vascularized ureteral margins [57].

**Fig. 6:**
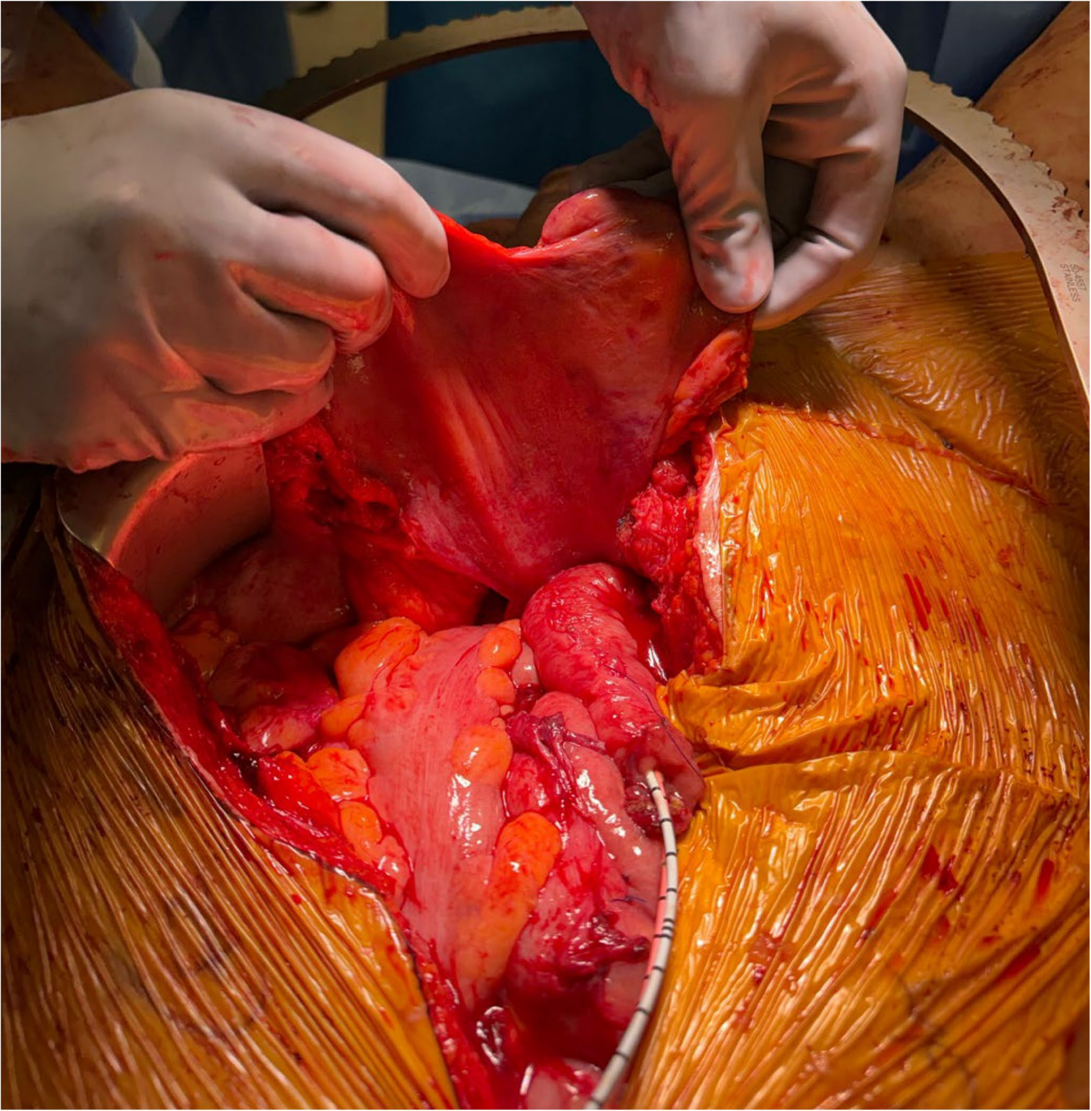
Peritoneal flap to cover pelvic defect following pubic bone debridement in osteomyelitis associated with urosymphyseal fistula

Vascularized flap coverage of the pubectomy site and obliteration of pelvic dead space is another essential portion of the procedure. The peritoneum can be carefully dissected away from the dome of the bladder for later use as a pelvic flap. Omental flaps are frequently mobilized on a right gastroepiploic pedicle to obliterate dead space, protect anastomotic sites, and cover exposed bony surfaces [2]. Finally, for those with extensive pelvic dead space or very large fistulae, rectus abdominus flaps can be harvested, often with the help of a plastic surgeon (Fig. 6, supplemental video 1).

Advancements in robotic-assisted surgery have paralleled trends in radical cystectomy for bladder cancer, offering reduced blood loss and shorter hospital stays [58, 59]. Emerging techniques, such as Holmium: YAG laser-assisted debridement, have been employed in select cases, allowing for precise ablation of necrotic cartilage and infected bone while preserving the anterior ligamentous support of the pubic symphysis [59, 60]. Similarly to open, Robotic-assisted USF and POM repair can benefit from NIFI/ICG by providing real-time visualization of tissue perfusion and anatomical landmarks, which aids in precise dissection and identification of pathological structures. This technique has been shown to improve outcomes in various robotic urological surgeries, such as ureteral reconstructions and partial nephrectomies, by enhancing the surgeon’s ability to assess vascularity and delineate healthy from diseased tissue [61–64]. With advancements in surgical techniques—such as the use of scaffolding tissue biografts to reduce anastomotic leak rates—salvage radical prostatectomy with redo vesicourethral anastomosis (VUA) may be considered when the prostate remains in situ [65]. However, this approach is generally discouraged due to the high risk of recurrent fistulization [66].

Postoperatively, prolonged antibiotic therapy remains critical, with a single-stage surgical success rate exceeding 80% in some series, underscoring the efficacy of aggressive but well-coordinated surgical management in these cases [67].

### Treatment Failure

Treatment failure is a complex clinical challenge and unfortunately not uncommon. Literature on medical-only management limited in patients with USF. Additionally, the initial data suggesting that medical-only management might be protective against failure in patients with non-USF POM must be interpreted with caution, as patients opting for medical-only management often have fewer risk factors for recurrence, such as fewer comorbidities, improved frailty scores, and less incidence of prior pelvic surgery [6, 13]. Risk factors for treatment failure of medical-only management include polymicrobial infections and antimicrobial-resistant strains, such as methicillin-resistant Staphylococci and extended-spectrum beta-lactamase-producing Enterobacteriaceae [6, 12, 68]. Medical-only management may be trialed in patients after appropriate counseling, with careful consideration of patient goals of care. It is critical that patients understand that medical-only management may resolve symptoms, however probability of recurrence and ultimate progression toward surgical management is likely; medical-only management may simply delay the inevitable.

Ultimately, a multidisciplinary approach combining medical optimization with intravenous antibiotics and surgical intervention — such as cystectomy with debridement or excision of the infected pubic symphysis — is believed to offer patients with these complex infections the best chance for successful treatment and reduced risk of recurrence. However, the risks associated with significant surgery must be carefully weighed, and patient selection is critical. Factors such as the patient’s overall health, the extent of infection, and previous treatments should be considered. Surgical debridement combined with pathogen-specific antibiotic therapy remains the cornerstone of treatment for infected pubic symphysitis with a USF [69].

Should a recurrence of POM occur following medical and surgical intervention for USF, interventions include prolonged courses of broad antibiotics including coverage for prior cultures and the potential need for additional surgical debridement. The use of antibiotic-impregnated resorbable biocomposites to fill resected space has been described in orthopedic journals for management of joint infections, although widespread use in the setting of POM with USF is not thoroughly described [70]. Management of recurrence following initial treatment ultimately must be personalized to the patient with careful consideration of patient health goals as well as their comorbidities and overall health status.

## Future Directions

While USF with POM remains a rare and debilitating condition, advancements in both diagnostic and surgical techniques are essential to improving patient outcomes. Delayed diagnosis continues to be a major challenge, with a median time of five months from symptom onset to definitive surgery [11, 56]. Future efforts should focus on early detection strategies, including refined imaging modalities and algorithm-driven diagnostic protocols, to expedite intervention and mitigate disease progression. Additionally, the incorporation of patient-reported outcomes into surgical decision-making is critical, as studies indicate that cystectomy is often the preferred approach, with a significant proportion of patients expressing no regrets postoperatively [11]. Improved quality of life and reduced postoperative narcotic use further support the necessity of refining surgical techniques to optimize recovery [71].

Looking ahead, the evolution of surgical management will likely emphasize minimally invasive techniques to reduce perioperative morbidity while maintaining functional efficacy. Emerging approaches, such as robotic-assisted cystectomy and urinary diversion and Holmium: YAG laser-assisted debridement, offer promising avenues for reducing blood loss, enhancing precision, and expediting recovery. Further research into tissue engineering and bioengineered grafts may also play a role in reconstructive strategies following pubic symphyseal resection [72]. Ultimately, a patient-centered approach, integrating technological advancements with improved perioperative care and rehabilitation, will be key to refining the future standard of care for POM from USF.

## Conclusion

**Fig. 7:**
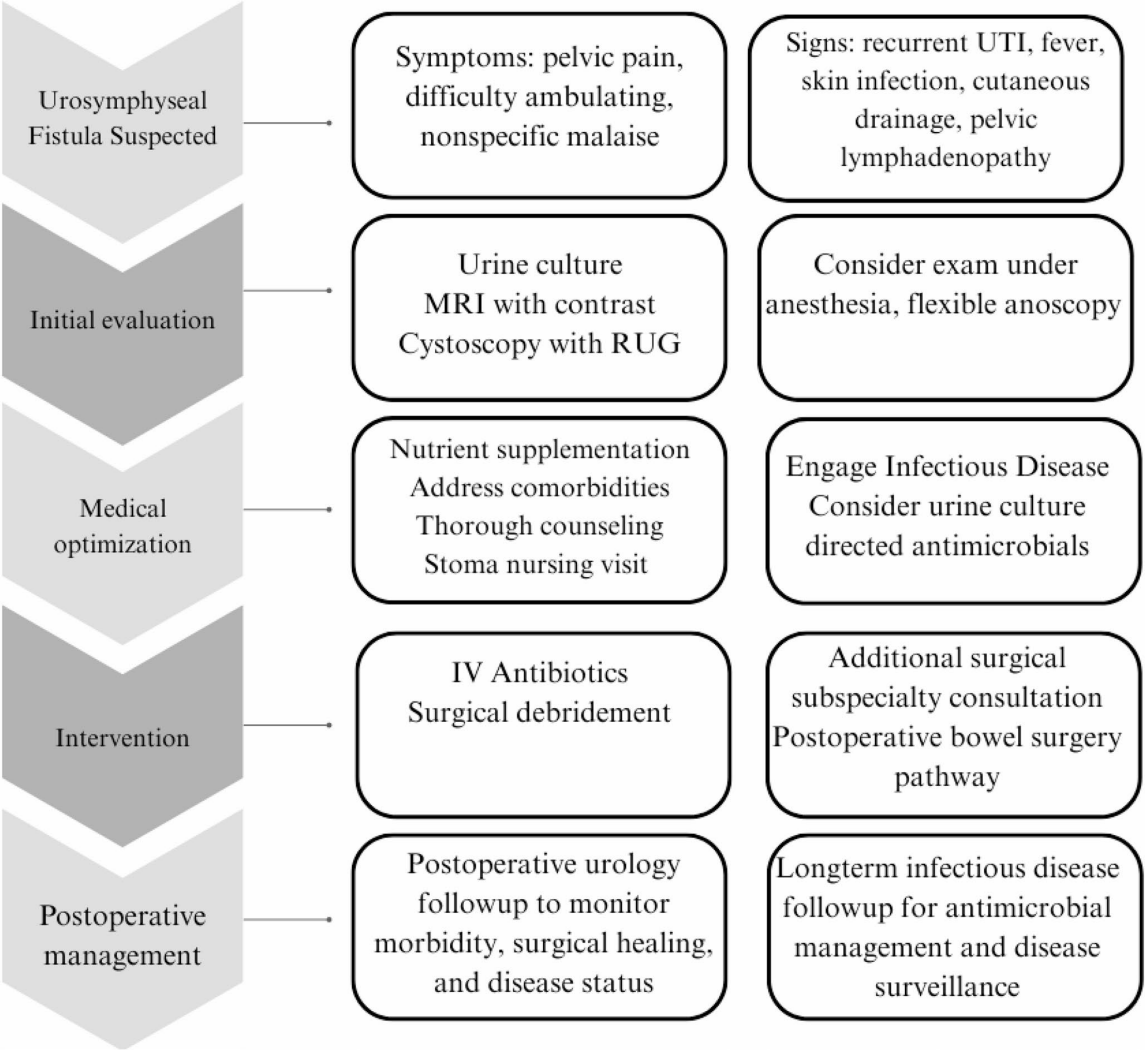
Proposed algorithm for management of urosymphyseal fistula

USF with POM is a rare and complex medical condition. Patient populations are often comorbid and have had prior pelvic radiotherapy in addition to urethral manipulation, making surgical interventions and wound healing more challenging. Diagnosis may be suspected based on symptoms and contrasted imaging studies, however definitive diagnosis should include cystourethroscopy, fluoroscopy, MRI, and ultimately pubic bone biopsy and culture. Management hinges on a multidisciplinary focus approach and includes recruitment of infectious disease specialists as well as various surgical subspecialists on a case-dependent bases. Medical optimization and counseling prior to extensive surgical intervention followed by long-term antibiotic regimens and infectious disease follow-up is critical to best positioning patients for success. Treatment failure with a purely nonsurgical approach is described and decision making for care requires careful consideration of patient symptoms and clinical status, comorbidities, prior pelvic interventions, and patient support systems. At our institution we approach USF and POM in a multidisciplinary fashion, which starts preoperatively and continues for years postoperatively (Fig. 7). Larger patient populations and longer follow-up time are needed to aid in the development of consensus diagnosis and management guidelines.

## Key References

- L. Smeyers, J. Borremans, F. Van der Aa, M. Herteleer, and S. Joniau, “The Complex Challenge of Urosymphyseal Fistula and Pubic Osteomyelitis in Prostate Cancer Survivors,” (in eng), Eur Urol Open Sci, vol. 70, pp. 43–51, Dec 2024, doi: 10.1016/j.euros.2024.09.008.
  - This study provides one of the most detailed institutional series on USF and pubic osteomyelitis in prostate cancer survivors, clarifying the strong association between prior pelvic radiotherapy, urethral manipulations, and the subsequent development of these complications, which often present years after initial cancer treatment. The findings reinforce that conservative management is rarely successful in irradiated patients, and that most require multidisciplinary surgical intervention and directly addresses gaps in the literature regarding optimal diagnostic and therapeutic strategies.
- U. A. Anele, H. M. Wood, and K. W. Angermeier, “Management of Urosymphyseal Fistula and Pelvic Osteomyelitis: A Comprehensive Institutional Experience and Improvements in Pain Control,” (in eng), Eur Urol Focus, vol. 8, no. 4, pp. 1110–1116, Jul 2022, doi: 10.1016/j.euf.2021.08.008.
  - This study represents one of the largest single-institution experiences with USF from 2009 to 2021, systematically characterizing clinical presentation, diagnostic delays, and the high prevalence of pelvic osteomyelitis in this population. The findings demonstrate that definitive surgical management—typically involving cystectomy, pubic bone debridement or resection, and soft-tissue flap reconstruction—results in significant reductions in pain scores and chronic opioid use, highlighting the importance of aggressive intervention for symptom control and quality of life.
- M. T. Walach, A. A. Tavakoli, G. Thater, M. C. Kriegmair, M. S. Michel, and M. C. Rassweiler-Seyfried, “Pubic bone osteomyelitis and fistulas after radiation therapy of the pelvic region: patient-reported outcomes and urological management of a rare but serious complication,” (in eng), World J Urol, vol. 42, no. 1, p. 461, Aug 01 2024, doi: 10.1007/s00345-024-05155-2.
  - By evaluating imaging progression and patient-reported outcomes, this study underscores the importance of integrating early radiological assessment and multidisciplinary care into post-radiation follow-up protocols, thereby informing future strategies for earlier detection, improved perioperative planning, and tailored management of USF and PO in high-risk populations.

## Supplementary Information

Below is the link to the electronic supplementary material.

Supplementary Material 1 (MOV 5.48 MB)

## Data Availability

No datasets were generated or analysed during the current study.
